# Genetic Overlap of Thoracic Aortic Aneurysms and Intracranial Aneurysms

**DOI:** 10.3390/genes16020154

**Published:** 2025-01-26

**Authors:** Mah I Kan Changez, Afsheen Nasir, Alexandra Sonsino, Syeda Manahil Jeoffrey, Asanish Kalyanasundaram, Mohammad A. Zafar, Bulat A. Ziganshin, John A. Elefteriades

**Affiliations:** Aortic Institute at Yale-New Haven Hospital, Yale School of Medicine, Yale University, New Haven, CT 06510, USA; mahikan.changez@yale.edu (M.I.K.C.);

**Keywords:** thoracic aortic aneurysm, intracranial aneurysm, ascending thoracic aortic aneurysm, genome-wide association studies, familial intracranial aneurysm (FIA)

## Abstract

Objective: Thoracic aortic aneurysms (TAAs) and intracranial aneurysms (ICAs) share overlapping genetic and pathophysiological mechanisms, yet the genetic interplay between these conditions remains insufficiently explored. This study aimed to identify common genetic factors underlying TAA and ICA. Methods: A comprehensive review of genome-wide association studies (GWASs) and retrospective clinical studies was conducted using PubMed, Orbis, and Web of Science. Articles addressing the genetic etiologies of TAA and ICA were analyzed. Separate lists of causative genes were compiled, and commonalities were identified. A Venn diagram was constructed to illustrate genetic overlap and shared physiological pathways. Results: We identified 24 overlapping genes associated with TAA and ICA, including *LTBP2*, *TGFB2*, *TGFB3*, *TGFBR1*, *TGFBR2*, *SMAD2*, *SMAD3*, *COL1A2*, *COL3A1*, *COL4A1*, *COL5A1*, *COL5A2*, *FBN1*, *FBN2*, *ELN*, *LOX ACTA2*, *MYH11*, *MYLK*, *ABCC6*, *NOTCH1*, *MED12*, *PKD1*, and *PKD2.* These genes are involved in pathways related to connective tissue biology, contractile elements, extracellular matrix components, and transforming growth factor-β signaling. While vascular endothelium and cell cycle pathways were unique to ICA, TAA pathways predominantly involved extracellular matrix remodeling. Conclusions: This study highlights the significant genetic overlap between TAA and ICA, shedding light on shared molecular mechanisms. These findings underscore the importance of interdisciplinary awareness: neurologists, neurosurgeons, and neurointerventional radiologists should monitor ICA patients for potential TAA, while cardiologists, cardiac surgeons, vascular surgeons, and vascular interventionalists should consider ICA risks in TAA patients. Further research into these genetic pathways could enhance the understanding and management of both conditions.

## 1. Introduction

Ascending thoracic aortic aneurysm (ATAA) is defined as aortic dilation in the aortic root and ascending aorta with diameters above 40 mm [1]. ATAA is considered an aggressive disease but an indolent one due to the thoracic aorta’s inherent nature of growing slowly—usually at less than 1 mm per year. Additionally, this disease’s long asymptomatic nature contributes to the perception of indolence [2]. Ascending thoracic aortic dissection (ATAD) is a lethal repercussion of ATAA, presenting as an acute delamination between the layers of the aortic walls (usually in the mid-media, between lamellar layers) [3]. Thoracic aortic aneurysm (TAA) is the 17th leading cause of mortality in patients over 65 years of age. Ruptured TAA carries a natural mortality rate of 94–100% [4]. Although the prevalence of TAA among the general population is difficult to ascertain due to its indolent nature, the literature estimates place the incidence and prevalence of TAA at 5.3 per 100,000 individuals/year and 0.16% of the population, respectively [5]. The incidence of ruptured aortic aneurysm is estimated at 1.6 per 100,000 [6].

Intracranial aneurysm (ICA) is characterized by an abnormal widening of the cerebral vessel lumen exceeding 50% of the normal size and all three vessel layers [7]. The prevalence of unruptured ICAs is estimated at 6.6% worldwide, with the minority of the ruptured ICAs causing subarachnoid hemorrhages (SAHs), estimated at 6 per 100,000 [7,8]. Among affected patients, 24% die before reaching the hospital. Overall, 39% of patients die within 1 month of rupture [9].

Given the similarities in the mechanism and human toll extracted by both TAA and ICA, we embarked on examining the medical literature to search for overlap of the causative genes of both the pathologies—TAA and ICA—which, to our knowledge, had not been specifically and rigorously performed before.

## 2. Methods

**Figure 1:**
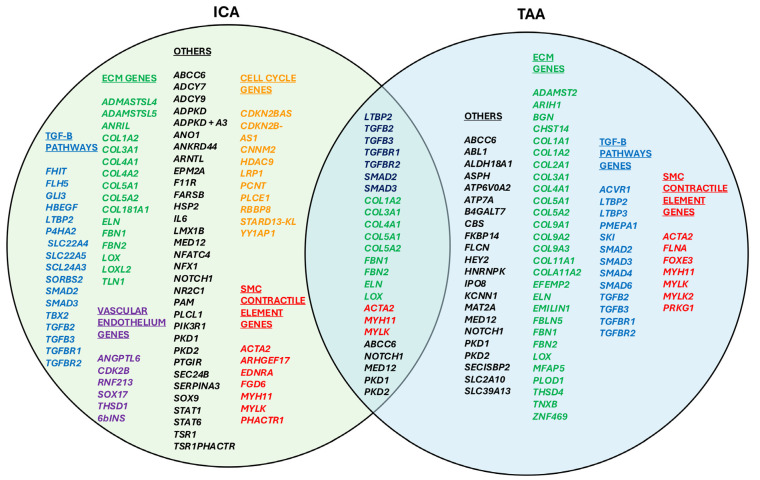
Venn diagram of genetic overlap between TAA and ICA with the genetic pathways color-coded, indicating each pathway as follows: green (extracellular matrix genes); blue (TGF-B pathways genes), red (SMC contractile element genes), brown (cell cycle genes), purple (vascular endothelium genes), and black (other pathways).

We performed an extensive literature review of multiple GWAS and other retrospective clinical studies. We scrutinized articles available on Pubmed, Web of Science, and Orbis databases. We searched keywords and MeSH terms pertinent to “Thoracic Aortic Aneurysm and Dissection”, “Intracranial Aneurysm”, and “genetics”. Also, specifically, TAA and ICA were searched in online genetic databases, such as Omim and ClinVar [10,11]. The selected gene variants that were identified and classified by the databases as ‘pathogenic’ or ‘likely pathogenic’ were chosen for incorporation in our study. The final scrutiny of the potential genetic variants was performed by devising comprehensive search strings with MeSH terms of each genetic variant on our list and its functional pathway with the diseases simultaneously. Articles highlighting causative variants for TAA and ICA were collected and scrutinized. Separate lists for causative agents were constructed for TAA and ICA, and then commonalities were sought. A Venn diagram was constructed to display the genetic overlap and common physiological pathways involved (Figure 1).

## 3. Results

### 3.1. TAA Genes

As per the American Association of Medical Genetics, a total of 70 genes have been identified to have a strong association with thoracic aortic aneurysm and dissection [12,13,14]. These can be classified according to the underlying function of the genes, such as extracellular matrix genes, smooth muscle contractile genes, transforming growth factor pathways, and other mechanisms. Also, some genes are *syndromic*, and some are *non-syndromic* [15,16].

**Figure 2:**
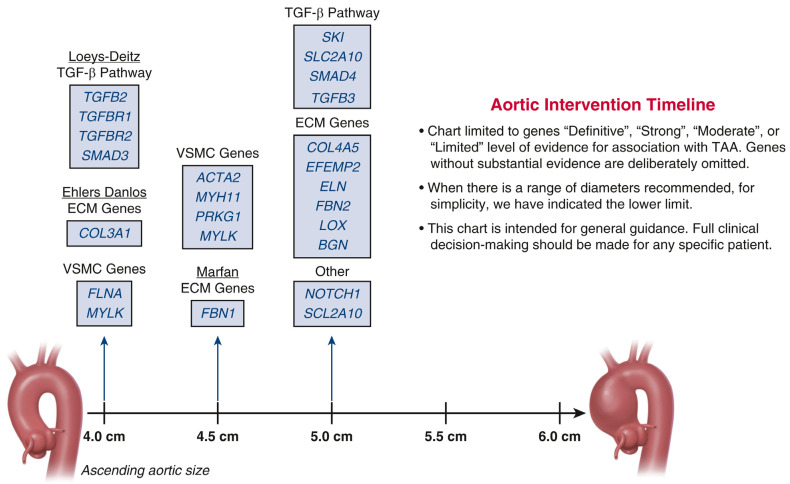
Timeline for surgical intervention for ascending thoracic aortic aneurysm (TAA). This chart lists the documented genetic mutations known to cause TAA. The arrows indicate the general aortic size threshold for surgical intervention in patients harboring each mutation. [Reprinted with permission from: Elefteriades, J.A.; Zafar, M.A.; Ziganshin, B.A. Genetics of aortic aneurysm disease: 10 key points for the practitioner. *JTCVS Open* 2024, 21, 58–63 [20]].

Syndromic TAAs manifest associated non-aortic abnormalities, such as skeletal, cutaneous, or facial characteristics. The most clinically significant syndromes include Marfan syndrome (with a mutation in the fibrillin-1 gene (*FBN1*)), Loeys–Dietz syndrome (with a mutation in transforming growth factor β 2 and 3 (*TGFB2*, *TGFB3*), transforming growth factor β type receptor 1 and 2 (*TGFBR1*, *TGFBR2*) and *SMAD2* and *SMAD3* genes). Lastly, Ehlers–Danlos syndrome results from a mutation in the collagen type III α 1 chain gene (*COL3A1*) [17,18,19]. Non-syndromic ATAAs result from mutations in a variety of genes and account for the majority of ATAD cases. Notably, non-syndromic subtype includes genetic mutations in myosin heavy chain 11 (*MYH11*), myosin light chain kinase (*MYLK*), neurogenic locus notch homolog protein 1 (NOTCH1), lysyl oxidase (*LOX*), actin α 2 (*ACTA2*), forkhead box E3 (*FOXE3*), and protein kinase cGMP-dependent 1 (*PRKG1*), amongst others. A “timeline” for surgical intervention has been established based on aortic sizes for these genes and updated recently by our team (Figure 2) [20].

### 3.2. ICA Genes

Using online genetic directories and databases, 88 genes were found to be associated with ICA [10,21,22]. The enlisted genes can also be subdivided as per their functional involvement; extracellular matrix, smooth muscle contractile element, transforming growth factor pathways, cell cycle, vascular endothelium, and others. Two major subtypes, familial versus sporadic, have been identified [23]. In familial ICAs (FIAs), evidence-based learning from population-based and case–control studies has shown that there are genetic factors contributing to the development and rupture of ICAs with a four-fold greater risk in first or second-degree relatives as compared to the general population. These familial aneurysm most commonly involve the middle cerebral artery [24,25,26]. On the other hand, sporadic ICAs have a proclivity to present with subarachnoid hemorrhages (SAH) at a younger age, to be multiple, and to involve the anterior communicating artery [27]. Of note, ICAs have been seen to have associations with monogenic disorders which are caused by penetrant mutations of a single gene, typically displaying Mendelian inheritance. Causative genes include autosomal polycystic kidney disease (*PKD1*, *PKD2*), Ehlers–Danlos syndrome (*COL3A1*), Marfan syndrome (*FBN1*), Loeys–Dietz syndrome (*TGFB2*, *TGFB3*, *TGFBR1*, *TGFBR2*, *SMAD2*, *SMAD3*), and Majewski Osteodysplastic Primordial Dwarfism (*PCNT*) [23].

### 3.3. Overlapping Genes

Although multitudes of genes play pivotal roles in the complex pathophysiology of TAA or ICA as separate entities, we were able to identify a definite, meaningful overlap of 24 independent genes based on genome-wide association studies or via other genetic methods. The overlapping genes are *LTBP2*, *TGFB2*, *TGFB3*, *TGFBR1*, *TGFBR2*, *SMAD2*, *SMAD3*, *COL1A2*, *COL3A1*, *COL4A1*, *COL5A1*, *COL5A2*, *FBN1*, *FBN2*, *ELN*, *LOX*, *ACTA2*, *MYH11*, *MYLK*, *ABCC6*, *NOTCH1*, *MED12*, *PKD1*, *PKD2*. The corresponding molecular pathways include contractile element genes, extracellular matrix genes, and transforming growth factor-β genes. Genes affecting vascular endothelium and cell cycle were unique to ICA.

The TAA and ICA overlapping genes are associated with several key biological pathways, primarily the transforming growth factor-β (TGF-β) signaling pathway, the extracellular matrix (ECM) organization, and Smooth Muscle Contraction and Cytoskeletal Organization:

- TGF-β Signaling Pathway: *TBP2*, *TGFB2*, *TGFB3*, *TGFBR1*, *TGFBR2*, *SMAD2*, *SMAD3*;
- Extracellular Matrix (ECM) Organization: *COL1A2*, *COL3A1*, *COL4A1*, *COL5A1*, *COL5A2*, *FBN1*, *FBN2*, *ELN*, *LOX*;
- Smooth Muscle Contraction and Cytoskeletal Organization: *ACTA2*, *MYH11*, *MYLK*.
- Other Pathways:
  ○ *ABCC6*: Encodes for a member of the ATP-binding cassette (ABC) transporter family, implicated in the transport of various molecules across membranes. Mutations in ABCC6 are associated with pseudoxanthoma elasticum, affecting elastic fibers in tissues.
  ○ *NOTCH1*: Part of the Notch signaling pathway, which influences cell fate decisions, proliferation, and apoptosis.
  ○ *MED12*: Encodes for a subunit of the Mediator complex, involved in transcriptional regulation by serving as a bridge between gene-specific transcription factors and the RNA polymerase II machinery.
  ○ *PKD1*, *PKD2*: Encode for polycystin-1 and polycystin-2, respectively, which are involved in calcium signaling pathways and are associated with autosomal dominant polycystic kidney disease.

## 4. Discussion

This review set out to identify any overlaps in causative genes of TAA with ICA, as well as to highlight any common genetic, biological processes pivotal for the pathophysiology of both these ailments.

Since the Human Genome Project began in 1990 [28], the advancement of bioinformatics tools used for genome-wide association studies (GWASs) has provided comprehensive insights into decoding the DNA that constitutes the human genome and clarifying the fundamental causation of numerous diseases.

Ascending thoracic aortic aneurysm (ATAA) is largely instigated by a single letter change in base or nucleotide. It is remarkable that a change in a single gene ‘letter’ among the 3.2 billion letters in the genome can cause such a devasting disease [15]. Multitudes of genetic-based studies, including GWAS, have been performed for TAA to highlight causative pathological genetic variants at numerous loci [29,30,31,32].

A recent study by Klarin et al. [33] conducted a GWAS on a cohort of 8626 patients with thoracic aortic aneurysm and dissection enrolled in the Million Veteran Program. Their results mirrored commonly known variants, including *FBN1*, *LRP1*, and *ELN*, and hypothesized the presence of 17 new risk loci associated with TAA. Additionally, recent studies also demonstrated a higher prevalence of variants of uncertain significance (VUS) compared with control genomes [34].

Interestingly, due to sex-related differences in cardiovascular diseases, cardiac involvement in female patients tends to be underdiagnosed, presenting later in life with a poorer prognosis [35,36,37]. A whole exome sequencing conducted by Chen et al. on a cohort of 179 Chinese females with thoracic aortic dissection evaluated 12 risk genes, including *ACTA2*, *COL3A1*, *LOX*, *MYLK*, *MYH11*, and resulted in the unveiling of 18 pathogenic or likely pathogenic variants [38].

Since its inception, GWAS investigations have also been widely conducted on ICA patients from diverse populations [39,40,41]. In an attempt to further characterize the genetic architecture, Bakker et al. [42] conducted a cross-ancestry GWAS meta-analysis with 10,754 ICA cases, which successfully recognized 17 risk loci. That study further emphasized a substantial single-nucleotide polymorphism (SNP) heritability of ICA, as previously shown in a twin study to be 41% [43].

To investigate phenotypical features in familial intracranial aneurysms (FIAs), a recent whole exome sequencing analysis enrolled 28 ICA patients from 26 Korean families with two or more first-degree ICA-affected family members [44]. Among 11 potential ICA-associated variants, the study noted three rare, potentially deleterious variants (*PLOD3*, *NTM*, and *CHST1*) in the subsets of FIAs in the cohort.

Another significant genome-wide linkage analysis study by Bakker et al. [45] conducted on three large Dutch families with multiple ICA-affected first-degree family members identified six rare damaging variants in genes: *SYCP1*, *FMNL2*, *TBC1D2*, *ZNF782*, *CCDC180*, and *NCBP1*.

Further GWAS for ICA have recognized potential variants involved in various biological pathways, such as NF-kB signaling, DNA methylation, matrix metalloproteinases, inflammation-related genes, and others [46,47,48,49].

Recognition of aggressive pathological genetic variants by GWAS has the potential to revolutionize the management of familial diseases with the prospect of leading to efficacious, biologically based medical therapies, especially when focusing on genetic overlaps between myriads of similar pathologies [50,51]. This can further enhance the screening process for overlapping pathologies as identifying ICAs in TAA patients can be very important, especially before the ascending aortic surgery; otherwise, intraoperative ICA rupture can occur with devastating consequences [52].

The substantial overlapping in genes causing TAA and those responsible for ICA are indicated in Figure 1. Indeed, our investigation has clearly shown that there is considerable overlap in the genes responsible for arterial aneurysms in two anatomically distant locations: the brain and the ascending thoracic aorta.

For the thoracic aorta, we utilize the “timeline” (Figure 2) graph for determining the most appropriate timing of prophylactic intervention in the setting of specific genetic aberrations. This is based on the premise that many genes alter the “normal” natural history of TAA by predisposing the patient to an earlier onset of aortic dissection or rupture and at smaller aortic sizes. Each mutation or variant in these genes affects the aortic wall differently, thereby creating a unique molecular subtype of disease for each gene. For example, mutations in genes within the TGF-β signaling pathway exhibit variable effects on aortic growth and remodeling. Thereby, mutations in *TGFBR1* and *TGFBR2* are associated with more aggressive aortopathy, often presenting with early-onset aortic dilation and a higher risk of dissection at smaller diameters, which may justify surgical intervention at 4 cm. This is thought to be due to heightened signaling perturbations leading to increased matrix degradation and vascular wall instability. Conversely, other TGF-β pathway mutations, such as those in *SMAD3*, may result in a more gradual progression of aortic dilation, leading to the standard surgical threshold of 5 cm. The differential impact on extracellular matrix remodeling, smooth muscle cell function, and vascular wall integrity could contribute to these distinct clinical phenotypes.

## 5. Conclusions

We have successfully identified an interplay of genes and their corresponding molecular pathways involved in the development of TAA and ICA. We hope these insights will lead to further clinical and scientific understanding of each disease via the study of their fundamental commonalities.

Most importantly, we call on neurologists, neurosurgeons, and neurointerventional radiologists to be aware that their ICA patients may well harbor TAAs. Concurrently, we call on cardiologists, cardiac surgeons, vascular surgeons, and vascular interventionalists to be aware that their TAA patients may well harbor ICAs. For each specialty, we need to be aware that aneurysms in the other zone—brain or aorta—may have just as heavy, if not even greater, impact on longevity and quality of life as the aneurysm in our zone of specialty.

## Data Availability

The data presented in this article are openly available on the omim and clinvar websites at (https://www.omim.org, 1 May 2024) and (https://www.ncbi.nlm.nih.gov/clinvar/, 1 May 2024).

## Abbreviations

TAA	Thoracic Aortic Aneurysm
ICA	Intracranial Aneurysm
ATAA	Ascending Thoracic Aortic Aneurysm
ATAD	Ascending Thoracic Aortic Dissection
FIA	Familial Intracranial Aneurysm
GWAS	Genome-Wide Association Studies

## References

[1] Johnston K.W., Rutherford R.B., Tilson M.D., Shah D.M., Hollier L., Stanley J.C. (1991). Suggested standards for reporting on arterial aneurysms. Subcommittee on Reporting Standards for Arterial Aneurysms, Ad Hoc Committee on Reporting Standards, Society for Vascular Surgery and North American Chapter, International Society for Cardiovascular Surgery. J. Vasc. Surg..

[2] Elefteriades J.A., Farkas E.A. (2010). Thoracic aortic aneurysm clinically pertinent controversies and uncertainties. J. Am. Coll. Cardiol..

[3] Shen Y.H., LeMaire S.A., Webb N.R., Cassis L.A., Daugherty A., Lu H.S. (2020). Aortic Aneurysms and Dissections Series: Part II: Dynamic Signaling Responses in Aortic Aneurysms and Dissections. Arterioscler. Thromb. Vasc. Biol..

[4] Ostberg N., Zafar M. Thoracic Aortic Aneurysms and Dissection. Encyclopedia. https://encyclopedia.pub/entry/11331.

[5] Melo R.G.E., Duarte G.S., Lopes A., Alves M., Caldeira D., e Fernandes R.F., Pedro L.M. (2022). Incidence and Prevalence of Thoracic Aortic Aneurysms: A Systematic Review and Meta-analysis of Population-Based Studies. Semin. Thorac. Cardiovasc. Surg..

[6] Bickerstaff L., Pairolero P., Hollier L., Melton L., Vanpeenen H., Cherry K., Joyce J., Lie J. (1982). Thoracic aortic aneurysms: A population-based study. Surgery.

[7] Rivera P.A., Dattilo J.B. (2024). Pseudoaneurysm. StatPearls.

[8] Etminan N., Chang H.-S., Hackenberg K., de Rooij N.K., Vergouwen M.D.I., Rinkel G.J.E., Algra A. (2019). Worldwide Incidence of Aneurysmal Subarachnoid Hemorrhage According to Region, Time Period, Blood Pressure, and Smoking Prevalence in the Population: A Systematic Review and Meta-analysis. JAMA Neurol..

[9] Asikainen A., Korja M., Kaprio J., Rautalin I. (2023). Case Fatality in Patients With Aneurysmal Subarachnoid Hemorrhage in Finland: A Nationwide Register-Based Study. Neurology.

[10] Home—OMIM. Omim.org. https://omim.org/.

[11] ClinVar. (n.d.). Nih.gov. https://www.ncbi.nlm.nih.gov/clinvar/.

[12] Milewicz D.M., Guo D., Hostetler E., Marin I., Pinard A.C., Cecchi A.C. (2021). Update on the genetic risk for thoracic aortic aneurysms and acute aortic dissections: Implications for clinical care. J. Cardiovasc. Surg..

[13] Brownstein A.J., Ziganshin B.A., Kuivaniemi H., Body S.C., Bale A.E., Elefteriades J.A. (2017). Genes Associated with Thoracic Aortic Aneurysm and Dissection: An Update and Clinical Implications. Aorta.

[14] Milewicz D.M., Regalado E.S., Shendure J., Nickerson D.A., Guo D.C. (2014). Successes and challenges of using whole exome sequencing to identify novel genes underlying an inherited predisposition for thoracic aortic aneurysms and acute aortic dissections. Trends Cardiovasc. Med..

[15] Jeoffrey S.M.H., Kalyanasundaram A., Zafar M.A., Ziganshin B.A., Elefteriades J.A. (2023). Genetic Overlap of Spontaneous Dissection of Either the Thoracic Aorta or the Coronary Arteries. Am. J. Cardiol..

[16] Czerny M., Grabenwöger M., Berger T., Aboyans V., Della Corte A., Chen E.P., Desai N.D., Dumfarth J., Elefteriades J.A., Etz C.D. (2024). EACTS/STS Guidelines for Diagnosing and Treating Acute and Chronic Syndromes of the Aortic Organ. Ann. Thorac. Surg..

[17] Dietz H.C., Cutting C.R., Pyeritz R.E., Maslen C.L., Sakai L.Y., Corson G.M., Puffenberger E.G., Hamosh A., Nanthakumar E.J., Curristin S.M. (1991). Marfan syndrome caused by a recurrent de novo missense mutation in the fibrillin gene. Nature.

[18] Gordon K.J., Blobe G.C. (2008). Role of transforming growth factor-β superfamily signaling pathways in human disease. Biochim. Biophys. Acta.

[19] Malfait F., Francomano C., Byers P., Belmont J., Berglund B., Black J., Bloom L., Bowen J.M., Brady A.F., Burrows N.P. (2017). The 2017 international classification of the Ehlers-Danlos syndromes. Am. J. Med. Genet. C Semin. Med. Genet..

[20] Elefteriades J.A., Zafar M.A., Ziganshin B.A. (2024). Genetics of aortic aneurysm disease: 10 key points for the practitioner. JTCVS Open.

[21] Gene Ontology Resource. https://geneontology.org/.

[22] Genecards.org. https://www.genecards.org/.

[23] Bakker M.K., Ruigrok Y.M. (2021). Genetics of Intracranial Aneurysms. Stroke.

[24] Wang P.S., Longstreth W.T., Koepsell T.D. (1995). Subarachnoid hemorrhage and family history. A population-based case-control study. Arch. Neurol..

[25] Schievink W.I., Schaid D.J., Michels V.V., Piepgras D.G. (1995). Familial aneurysmal subarachnoid hemorrhage: A community-based study. J. Neurosurg..

[26] Ronkainen A., Hernesniemi J., Puranen M., Niemitukia L., Vanninen R., Ryynänen M., Kuivaniemi H., Tromp G. (1997). Familial intracranial aneurysms. Lancet.

[27] Slot E.M.H., Rinkel G.J.E., Algra A., Ruigrok Y.M. (2019). Patient and aneurysm characteristics in familial intracranial aneurysms. A systematic review and meta-analysis. PLoS ONE.

[28] Collins F.S., Fink L. (1995). The Human Genome Project. Alcohol. Health Res. World.

[29] LeMaire S.A., McDonald M.-L.N., Guo D.-C., Russell L., Miller C.C., Johnson R.J., Bekheirnia M.R., Franco L.M., Nguyen M., Pyeritz R.E. (2011). Genome-wide association study identifies a susceptibility locus for thoracic aortic aneurysms and aortic dissections spanning FBN1 at 15q21.1. Nat. Genet..

[30] Guo D.-C., Grove M.L., Prakash S.K., Eriksson P., Hostetler E.M., LeMaire S.A., Body S.C., Shalhub S., Estrera A.L., Safi H.J. (2016). Genetic Variants in LRP1 and ULK4 Are Associated with Acute Aortic Dissections. Am. J. Hum. Genet..

[31] Xu H., Chen S., Zhang H., Zou Y., Zhao J., Yu J., Le S., Cui J., Jiang L., Wu J. (2020). Network-based analysis reveals novel gene signatures in the peripheral blood of patients with sporadic nonsyndromic thoracic aortic aneurysm. J. Cell. Physiol..

[32] Guo D.-C., Hostetler E.M., Fan Y., Kulmacz R.J., Zhang D., Nickerson D.A., Leal S.M., LeMaire S.A., Regalado E.S., Milewicz D.M. (2017). Heritable Thoracic Aortic Disease Genes in Sporadic Aortic Dissection. J. Am. Coll. Cardiol..

[33] Klarin D., Devineni P., Sendamarai A.K., Angueira A.R., Graham S.E., Shen Y.H., Levin M.G., Pirruccello J.P., Surakka I., Karnam P.R. (2023). Genome-wide association study of thoracic aortic aneurysm and dissection in the Million Veteran Program. Nat. Genet..

[34] Mahlmann A., Elzanaty N., Saleh M., Irqsusi M., Rastan A., Leip J.L., Behrendt C.-A., Ghazy T. (2024). Prevalence of Genetic Variants and Deep Phenotyping in Patients with Thoracic Aortic Aneurysm and Dissection: A Cross-Sectional Single-Centre Cohort Study. J. Clin. Med..

[35] Asatryan B., Yee L., Ben-Haim Y., Dobner S., Servatius H., Roten L., Tanner H., Crotti L., Skinner J.R., Remme C.A. (2021). Sex-Related Differences in Cardiac Channelopathies: Implications for Clinical Practice. Circulation.

[36] Smedberg C., Steuer J., Leander K., Hultgren R. (2020). Sex differences and temporal trends in aortic dissection: A population-based study of incidence, treatment strategies, and outcome in Swedish patients during 15 years. Eur. Heart J..

[37] Rieß H.C., Debus E.S., Schwaneberg T., Sedrakyan A., Kölbel T., Tsilimparis N., Larena-Avellaneda A., Behrendt C.-A. (2019). Gender disparities in fenestrated and branched endovascular aortic repair. Eur. J. Cardiothorac. Surg..

[38] Chen Y., Wang L., Xu X., Li K., Sun Y., Wang Y., Wang D.W. (2023). Genetic architecture of thoracic aortic dissection in the female population. Gene.

[39] Bilguvar K., Yasuno K., Niemelä M., Ruigrok Y.M., Fraunberg M.U., van Duijn C.M., Berg L.H.D., Mane S., Mason C.E., Choi M. (2008). Susceptibility loci for intracranial aneurysm in European and Japanese populations. Nat. Genet..

[40] Yasuno K., Bilguvar K., Bijlenga P., Low S.-K., Krischek B., Auburger G., Simon M., Krex D., Arlier Z., Nayak N. (2010). Genome-wide association study of intracranial aneurysm identifies three new risk loci. Nat. Genet..

[41] Yasuno K., Bakırcıoğlu M., Low S.-K., Bilgüvar K., Gaál E., Ruigrok Y.M., Niemelä M., Hata A., Bijlenga P., Kasuya H. (2011). Common variant near the endothelin receptor type A (EDNRA) gene is associated with intracranial aneurysm risk. Proc. Natl. Acad. Sci. USA.

[42] Stroke H.A.-I., Bakker M.K., van der Spek R.A.A., van Rheenen W., China Kadoorie Biobank Collaborative Group, BioBank Japan Project Consortium, The ICAN Study Group, CADISP Group, Genetics and Observational Subarachnoid Haemorrhage (GOSH) Study investigators, International Stroke Genetics Consortium (ISGC) (2020). Genome-wide association study of intracranial aneurysms identifies 17 risk loci and genetic overlap with clinical risk factors. Nat. Genet..

[43] Korja M., Silventoinen K., McCarron P., Zdravkovic S., Skytthe A., Haapanen A., de Faire U., Pedersen N.L., Christensen K., Koskenvuo M. (2010). Genetic epidemiology of spontaneous subarachnoid hemorrhage: Nordic Twin Study. Stroke.

[44] Song Y., Lee J.K., Lee J.O., Kwon B., Seo E.J., Suh D.C. (2022). Whole Exome Sequencing in Patients with Phenotypically Associated Familial Intracranial Aneurysm. Korean J. Radiol..

[45] Bakker M.K., Cobyte S., Hennekam F.A.M., Rinkel G.J.E., Veldink J.H., Ruigrok Y.M. (2022). Genome-wide linkage analysis combined with genome sequencing in large families with intracranial aneurysms. Eur. J. Hum. Genet..

[46] Shima Y., Sasagawa S., Ota N., Oyama R., Tanaka M., Kubota-Sakashita M., Kawakami H., Kobayashi M., Takubo N., Ozeki A.N. (2023). Increased PDGFRB and NF-κB signaling caused by highly prevalent somatic mutations in intracranial aneurysms. Sci. Transl. Med..

[47] Maimaiti A., Turhon M., Abulaiti A., Dilixiati Y., Zhang F., Axieer A., Kadeer K., Zhang Y., Maimaitili A., Yang X. (2023). DNA methylation regulator-mediated modification patterns and risk of intracranial aneurysm: A multi-omics and epigenome-wide association study integrating machine learning, Mendelian randomization, eQTL and mQTL data. J. Transl. Med..

[48] Kim B.J., Hong E.P., Youn D.H., Jeon J.P., First Korean Stroke Genetics Association Research (2022). Genome-Wide Association Study of the Relationship Between Matrix Metalloproteinases and Intracranial Aneurysms. J. Clin. Neurol..

[49] Hong E.P., Cho S.M., Rhim J.K., Park J.J., Ahn J.H., Youn D.H., Kim J.-T., Park C.H., Lee Y., Jeon J.P. (2023). Updated Trans-Ethnic Meta-Analysis of Associations between Inflammation-Related Genes and Intracranial Aneurysm. J. Korean Neurosurg. Soc..

[50] Gyftopoulos A., Ziganshin B.A., Elefteriades J.A., Ochoa Chaar C.I. (2023). Comparison of Genes Associated with Thoracic and Abdominal Aortic Aneurysms. Aorta.

[51] Brownstein A.J., Bin Mahmood S.U., Saeyeldin A., Velasquez Mejia C., Zafar M.A., Li Y., Rizzo J.A., Dahl N.K., Erben Y., Ziganshin B.A. (2019). Simple renal cysts and bovine aortic arch: Markers for aortic disease. Open Heart.

[52] Malhotra A., Seifert K., Wu X., Matouk C., Elefteriades J.A. (2019). Screening for Intracranial Aneurysms in Patients with Thoracic Aortic Aneurysms. Cerebrovasc. Dis..

